# Our Berlin Letter

**Published:** 1891-12

**Authors:** T. M. McIntosh

**Affiliations:** Thomasville, Ga.


### OUR BERLIN LETTER.

BERLIN, September, 1891.

*Editor Southern Medical Record:*

The two most careful men in the appointments of their operating rooms, cleanliness of instruments, preparation of patients, etc., are Leopold, the gynecologist, of Dresden, and Hirschberg, the oculist, of Berlin. The former, however, is lavish in the use of bichloride for hands and patient disinfection, while the latter believes that bichloride is not necessary to perfect asepsis. In the operation room of each every visitor wears a white sterilized operating apron. Hirschberg even goes so far as to require that those who witness his cataract and other internal eye operations shall not have been in the dissecting or pathological room for ten days. He takes all precautions that the gynecologist does with his laparotomies, and his operating room has the glass and metal table, tiled floor and stone walls of the laparotomist.

The day before a cataract operation, the patient has a complete bath, and is clothed in a suit thoroughly cleansed. The morning of the operation the entire face is thoroughly washed and then bathed with a 1-5000 solution bichloride mercury, but the conjunctiva is washed only with sterilized water. No bichloride solution ever comes on the conjunctiva. His instruments, sterilized after each operation, are again boiled before the next one, and cotton and gauze also are re-sterilized (I mean in a straw sterilizer). All of this preparation takes place under his own eye, and much is done by himself. He operates with the narrow knife, makes the upward incision, and usually in corneo-sclerotic juncture, and without iridectomy, as most of the leading operators do. His last 180 extractions gave only a loss of one case, which is phenomenally good. He uses silk in his muscle and conjunctival operations. For sympathetic ophthalmia, he yet makes, sometimes, a neurectomy, and for painful bulb, exsection of the optic nerve. In detachment of the retina, he obtains, in many cases, a normal visual field by the use of pilocarpine injections, pressure, and in some cases a puncture of the sclerotic. As interesting only from its novelty, and also from a diagnostic view, he showed in his clinics a case of vaccine of the eyelid which had been conveyed to the mother by the hand of her child, which had a typical pustule on its arm. He used instillations of eserine and atropine indefinitely without conjunctival irritation, and claims that when it does irritate the solutions are not properly sterilized.

Distilled water is the solvent for every eye lotion, and before used is resterilized, which is repeated every few days against infection which may occur after use.

No one in Berlin is such a worker. He takes no vacation. Each day every patient of his very large clinic passes under his eye, a careful record of which is kept, and being a most careful and correct observer, such vast experience must give him a wonderful fund of information, and make him a most excellent teacher. His sense of professional courtesy is very high, and he is very attentive to all physicians, and it seems to me, especially so to Americans, and he speaks English quite well.

Schwegger, the successor to von Graefe and the professor in the University, is the other eminent oculist here. As every one knows, he is a wonderfully rapid operator, and uses a modified Beer's knife. One of his assistants, Dr. Selig, also is a magnificent and rapid operator, and being as well a man of brains, must in the not distant future be well known.

Schwegger uses more bichloride than Hirschberg. The night before the extraction of a cataract, the eyelids and surrounding skin are well washed with soap and water and ether and a 1-5000 solution bichloride mercury, with which last solution the conjunctiva is also thoroughly cleansed. The eye is well bandaged until the following morning, and just before the operation it is rewashed with the sublimate solution. Gauze and bandages are thoroughly sterilized. Hands are washed in the 1-5000 sublimate solution, and the instruments boiled in a weak solutionof carbolic acid. To hold the lids, two long-handled Desmoore's lid-holders are used, and to fix the ball a two-pointed spear, each with shoulder to prevent too deep penetration into the sclerotic coat. Both of these individualisms are worthy of adoption, as there is no pressure on the ball with speculum or catch-forceps, and both can be rapidly removed and replaced in case of accident or other need. He makes the downward incision for the right eye and the upward for the left, as a matter of convenience, and directly in the corneo-sclerotic junction, and no iridectomy. This is the usual operation. As the case demands, he uses a different knife, incises in different parts of, and in different angles to the planes of the eye-ball, and makes an iridectomy. He operates in the ward room, where the patient remains during the time of treatment, and any one can witness it, as no special requirements are exacted. Portions of lens substance and capsules that do not come out readily, he removes with a small spoon introduced into the anterior chamber, and makes no rotary motion of the lids on the ball, with the finger, to bring the debris in the center of the pupil, and by upward pressure with the lid to reverse it, as some of the not very old books advise.

For sympathetic ophthalmia, he never makes neurectomy, as he says the nerve will again grow in a few weeks, but resects the optic nerve. He makes euncleations oftener than Hirschberg. He also, as does Hirschberg, makes a large iridectomy for glaucoma, and never sclerectomy, so popular for a short time a few years ago. The results of his extractions of 450 cases is a "loss" of twenty, which is a little over 4 per cent. while Hirschberg's is about one-third of one per cent. This is a remarkable difference.

Schwegger uses more antiseptics, but his rooms are not so clean. Hirschberg uses no antiseptics in the eye, but his operating room is faultlessly and painfully clean. Hirschberg did not give me, however, the result of his total, but said this loss of one had been in his last 180 operations. The method of dressing and after treatment of both are the same in so far that both use sterilized gauze, large amounts of sterilized cotton, secured with a roller bandage. Hirschberg's dressing is dry; Schwegger, however, washes the conjunctiva with a 1-5000 sublimate solution, and with the same wets the gauze which he places next the eye. Usually, after two days the first dressing is removed, afterwards daily; but the eye is not inspected unless there are evidences of inflammation, until about the sixth day. The technique of the operation seems to be, therefore, much a matter of habit, and the special incision is determined also by the custom of an individual operator, and what the special case to be operated on demands. First of all, however, the incision should be large enough. A too small incision is a most disastrous fault. This rule applies equally to all distinct methods and modifications of them. For those operators, however, whose experience is not vast, it would seem that the incision with the narrow knife, directly in corneo-sclerotic junction and an iridectomy, is the operation most worthy of general adoption. This minimizes the usual difficulties, the danger from loss of vitreous, and the liability to mal-nutritious slough, prolapse of the iris and its results. The limited experience of the writer, of thirty extractions, with different knives and different incisions, with his observations here, leads to the conclusion that the operations thus made will yield the most satisfactory results. With a Beer's knife, if the counter-puncture is too deep or too shallow, and the angle of the knife not correct, it cannot be remedied, while it can with a narrow knife, and the operation be completed satisfactorily, by a deeper or more forward turning of the incision. Once the Beer's knife was my preference To illustrate : Knapp, of New York, once made the puncture and counter-puncture with the cutting edge of the knife downward, which being discovered, he turned the blade in situ and satisfactorily finished the operation.

It is, perhaps, worth while to mention that no one here has adopted the method of dressing the eye so ably advocated by Dr. Chisholm, of Baltimore. He argues that the eye, in its normal state, is accustomed to light and has but the covering of the lid ; and that after operations these conditions should, as nearly as possible, be maintained ; and that the gauze and cotton dressing, dark room and recumbent position are unnecessary discomforts to the patient.

In dressing an amputated finger, it is not thought in the least that because it is normally without a covering, that it should have none. When wounded, it is in an *abnormal* state, and should be placed in reference to atmospheric temperature and immobility as nearly as possible in the condition of a subcutaneous injury, and should therefore have a good and bountiful aseptic covering.

When Lister, some fifteen years ago, gave to the world his method of wound-dressing, it seems to me that one of the details that will remain is the good of an abundant wound-covering. An eye is in an abnormal state after a cataract extraction, and is reasonably not exceptional. As to the recumbent position, in all probability two or three days are sufficient. This long, during which some union has occurred, great quiet is needed, and possibly can be better obtained recumbent than otherwise. There can be no question that any straining movements can produce great pressure from within the eye. Before the days of cocaine the writer gave chloroform to a nervous pat.ent to make an extraction. It was done nicely and without fault; all instruments were removed and the dressing was on hand to apply when the patient vomited and forced out vitreous.

In the University ear clinic one can almost daily witness the operation of trepanning the mastoid cells, which is done with a small ear chisel and mallet, the trephine never being used. The conditions requiring the operation are usually those of acute suppuration otetis media, with extension into the mastoid cells. Sometimes it is done where there is a compartively free discharge through the external auditory canal. The diseased mastoid cavity is usually well scraped out with the sharp bone spoon, and the wound dressed from the bottom with iodoform gauze. Dr. Janson is the assistant here, and during August and September, when I visited the clinic, was the operator. On August 15th he made a very pretty operation for abscess of the brain following acute mastoid inflammation, the diagnosis being made before the operation. The squamous portion of the temporal bone was trephined and about a teaspoonful of pus was evacuated by a puncture to the depth of half an inch into the brain substance two weeks before the mastoid had been opened and pus found. The symptoms that led to the diagnosis of brain abscess were: hemianopsia pain, paresis of right side (left ear diseased). The patient made a good recovery, and at the expiration of a month has almost entirely recovered from all symptoms of paresis. Your subscriber thinks that perhaps this operation is made here oftener than in America, and in conditions of less pressing and pronounced need. In children with acute otetis media, with development of brain symptoms, writers on ear disease claim that a faulty diagnosis is made often, and that death occurs from non-operative interference. When there is acute otetis media, and where there is great pain and prolonged suffering, even though no brain symptoms are present, it is better to err on the side of performing the operation too often than otherwise. It is an operation devoid of danger, almost, and the patient cannot be made worse by it. Your subscriber once made the operation with good results on an adult, and regrets that he did not more persistently insist on it in the case of a baby that had acute otetis media and died, but with not well defined brain symptoms

Perhaps if one should visit Berlin and not see much of von Bergman, the leading surgeon of Germany, and learn of his operations and his methods, the visit would be incomplete. As to any specially new operation and treatment of any special disease, I can say nothing of practical interest, but will give you the practical points of his wound treatment.

In a lecture to his private class in operative surgery, Schimmilbusch, his first assistant, said in substance: We are drifting further and further from antisepsis, and more and more toward asepsis. It is mechanical contact of decaying and impure substances that we must guard against, and not the atmosphere, as the medium of wound infection.

Instruments must, first of all, be mechanically cleaned, and then boiled for five minutes. To prevent them from rusting, which he claims is quite efficient, and also quite new, he puts in the water carbonate of soda to the amount of a five per cent. solution. Gauze, cotton bandages are placed for half an hour in a steam sterilizer, with double cylinders—that is, one cylinder within another and the bandage material in the internal one—and so fixed that the steam is under pressure. He impregnates his gauze with sublimate, boracic acid and iodoform. The two first are made in solutions, the bandage material wetted in it, and then dried. The iodoform he puts in dry, just as plaster of paris is put in mull for a plaster bandage.

For the ordinary closed wounds, he uses only the sterilized material as a dressing. For certain exposed wounds, or rather wounds which for various reasons require to be dressed daily, which, for purposes of inspection or proper healing need to be kept open, he uses iodoform gauze stuffed into them. His silk is boiled each time before using. His catgut is put in alcohol 80 per cent. strength, with sublimate to amount of 1 per cent solution of same. This is changed daily until the alcohol ceases to become cloudy. When prepared in this manner, it can be kept indefinitely in the alcohol, ready for use.

The brushes which he uses to cleanse the hands, and on the parts of the patient to be operated on, he boils, or as he says, "cooks," for half an hour before using. In the intervals of non-use, they are kept in a 1-2000 sublimate solution, which is often changed. Patient and hands are thoroughly washed in sol. of above strength before an operation, though he does not consider it necessary for the operator's hands. I have not spoken of sponges, for he uses instead the non-medicated sterilized gauze, and of course only once. However, the preparation and sterilization of sponges, which most physicians outside of large hospitals will use, is a matter of some practical as well as economic importance. Dr. Hahn, of Friedrickham hospital, washes his sponges that have been used, or that are new, and puts them in a 20 per cent. solution carbolic acid. Each day they are removed, rewashed and put again in a fresh carbolic solution. This is done for a week, and the sponges washed and sterilized by steam or boiling water, are ready for use.

Every physician can make his gauze and absorbent cotton for the ordinary use of cleansing the mucous membranes and suppurating wounds and ulcers; applications to the nose, throat and vagina. The ordinary cheese-cloth of the stores and the lint cotton of the farmers can be boiled in a weak solution of caustic soda, washed and reboiled in same solution, and rewashed until the oil, which prevents all new cotton material from absorbing readily, is thoroughly removed. For use in wounds this gauze and cotton should be exposed to steam sterilization, the apparatus for which, complete, is bulky and somewhat expensive, as your correspondent knows by the purchase of certain parts, not so bulky, the additions to which can be made at home and the instruments completed.

T. M. McIntosh, Thomasville, Ga.

